# Supplementary material for: Policy Implications for Protecting Health from the Hazards of Fire Smoke. A Panel Discussion Report from the Workshop Landscape Fire Smoke: Protecting Health in an Era of Escalating Fire Risk
Source: Int J Environ Res Public Health. 2021 May 26;18(11):5702. doi: 10.3390/ijerph18115702 (PMC8199356; doi:10.3390/ijerph18115702)
Supplement: Supplementary file 1 [file ijerph-18-05702-s001.zip › ijerph-1205569-supplementary.pdf]

Supplementary information

Material S1

Audience polling questions

The workshop participants were polled on their two preferred methods of protection during an elevated smoke event. The list of actions were adopted from advice made available by agencies during bushfire or controlled burning episodes.

1. What do you think are the most important ways to protect your health during extreme smoke events? If you were only allowed two strategies what would they be?

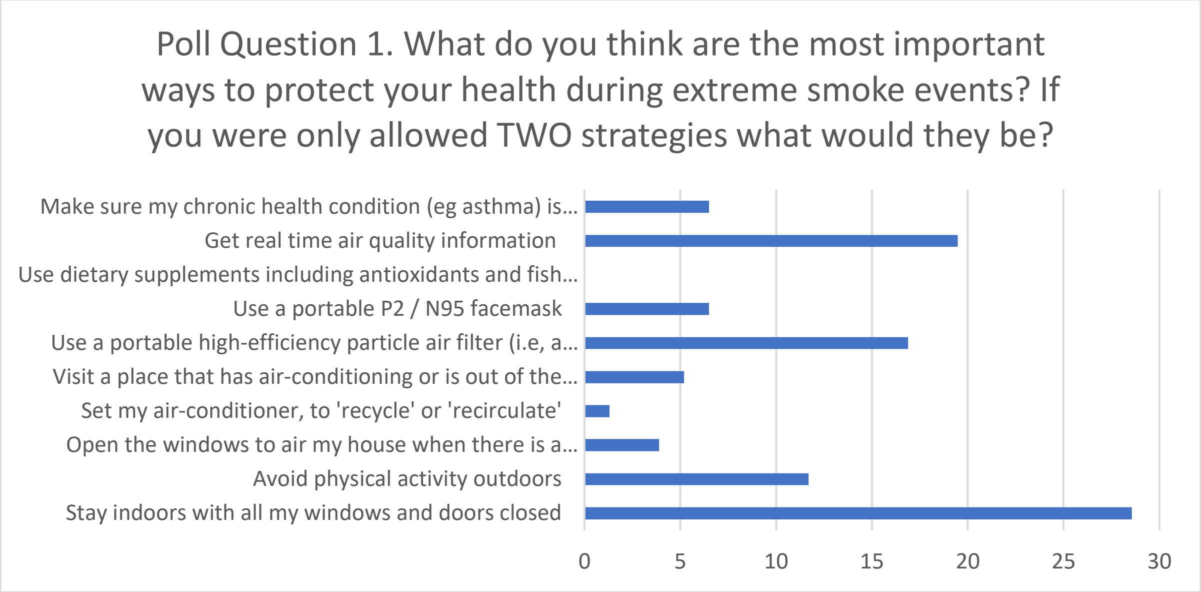

The highest scoring action chosen by the workshop participants was to *"stay indoors with doors and windows closed"* (29%). This was followed by 19% of participants indicating they would obtain *"real time air quality information*", and 17% of participants who would *"use a portable high-efficiency particle air filter (HEPA filter)"*. 12% of participants indicated that they would *"avoid physical activity outdoors"* and 6% indicated they would ensure their *"chronic health condition (e.g., asthma, cardiovascular disease) is well under control"*, and 6% indicated they would *"use a P2/N95 facemask"* (Figure 1).

2. Would you be comfortable with your tax dollars being used to subsidise the cost of air cleaners for people in higher risk groups for air pollution?

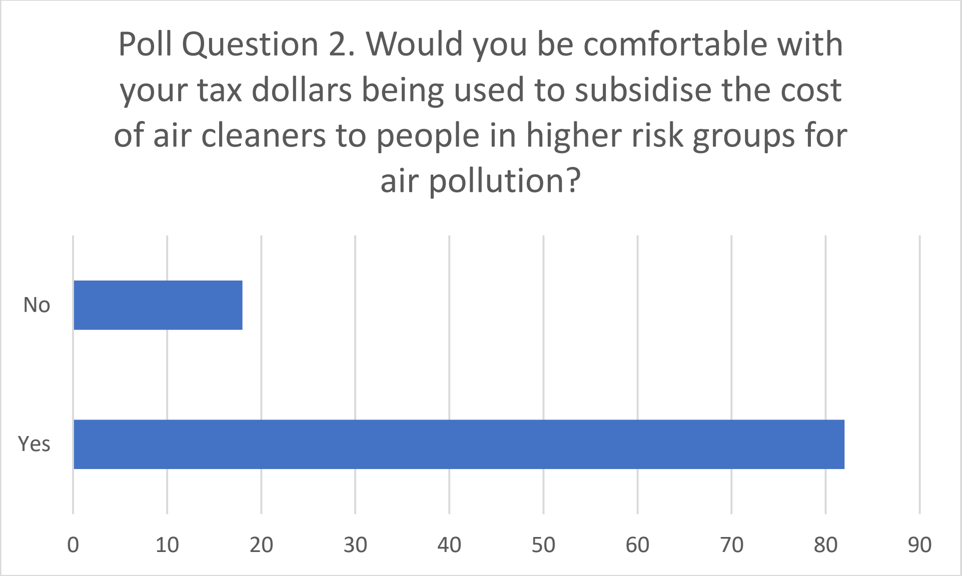

Audience participants were asked if they would be comfortable to use taxes to subsidise the cost of air cleaners for higher risk vulnerable groups. By far, the majority of participants (82%) indicated that they would support government subsidisation of air cleaners for this group.

3.   The bushfire inquiries have made recommendations on improvements in air quality reporting and the definition of air quality categories. What do you see as the highest priority group for use?

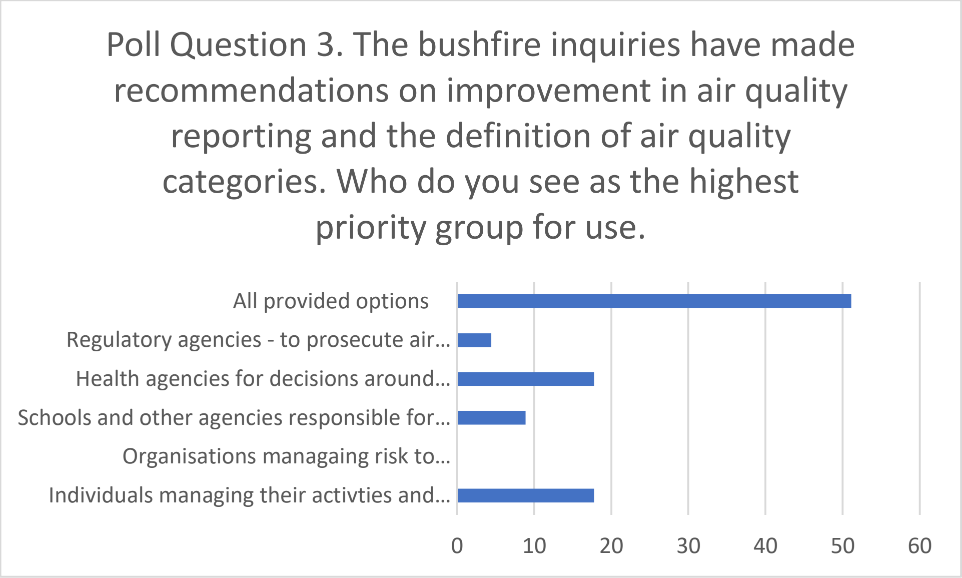

51% of participants responded that all the listed groups were high priority, 18% responded that the information individuals were the highest priority group to help manage their activities and health, 18% thought that health agencies were the greatest priority to help in decision making around messaging, and 9% responded that schools and agencies responsible for vulnerable population groups were of highest priority. The fact that no respondents chose organisations managing employees is likely to reflect the lack of participants from organisational associations or unions (1%).

4. Which areas of research would you recommend that the government focus providing funding for?

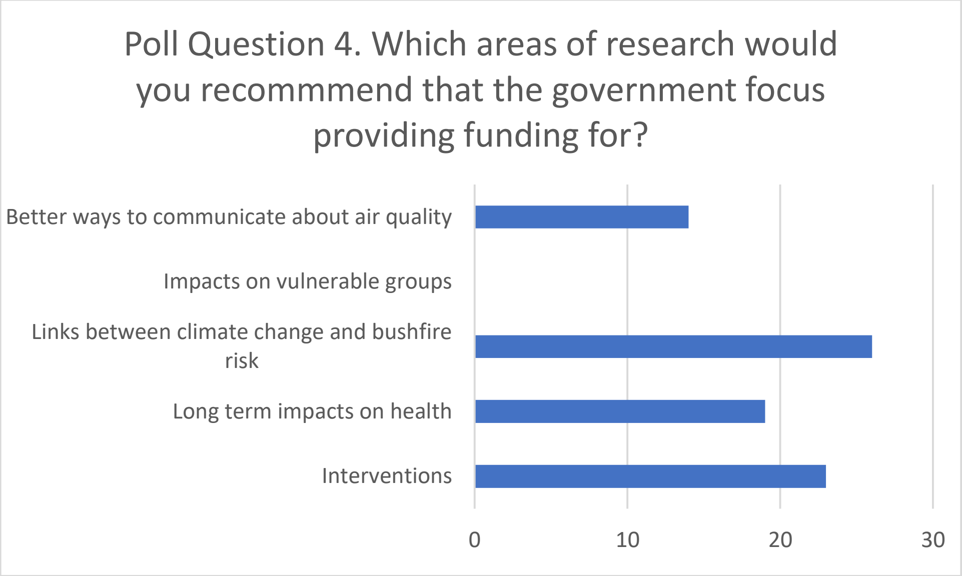

Workshop participants were polled on their preference for future priorities for government funding on bushfire smoke and health impact mitigation. 33% of participants indicated that government funding should be prioritised to be spent on *"better ways to communicate about air quality"*, followed by 26% voting for funding on the *"links between climate change and bushfire risk"*, 23% on *"interventions"* and 19% on the *"long term impacts on health*". Interestingly, and perhaps arguably, no participants thought that the government should prioritise spending on the impacts on vulnerable groups.

Material S2

Expert Panel Members

Michele Goldman, CEO Asthma Australia (representing an NGO and consumer advocacy group)

Asthma Australia is an advocacy group which provides direct support to the public by disseminating information to health professionals, through community organisations such as schools, and to the wider community through direct phone line services and its website. Asthma Australia also funds and commissions research, and conducts advocacy work, primarily aimed at diagnosis, treatment and avoidance of asthma. Asthma Australia is interested in air pollution exposures given they are a recognised trigger for asthma symptoms. Asthma Australia also helps to translate evidence into practice. Ms Goldman was asked as a panel member to contribute her expertise in advocacy, translation of science for public uptake and public education programs. Ms Goldman had met with government representatives and had made submissions to the bushfire inquiries. Asthma Australia also conducted an online survey of over 12,000 respondents on the health effects experienced during the 2019-2020 bushfire season ().

Professor Sotiris Vardoulakis, Professor of Global Environmental Health, Australian National University (ANU) (representing research and academia)

Professor Vardoulakis is a Professor of Global Health at the Australian National University's National Centre for Epidemiology and Population Health. He is founding co-chair of the International Consortium for Urban Environmental Health and Sustainability (Healthy-Polis). Over the last two decades, Professor Vardoulakis has advised national and local governments and international organizations, such as the World Health Organization and the European Parliament, on the health effects of climate change and air pollution, and on environmental sustainability and urban health. His main research interests include climate change and health, air pollution and health, sustainable cities, exposure assessment, epidemiology, health impact assessment, and environmental public health communication and policy. Professor Vardoulakis was asked as a panel member to contribute his expertise in scientific environmental health research and his experiences in preparing and communicating public health advice during the 2019-2020 bushfire season.

Dr Melissa Sweet, Croakey Health Media

Dr Melissa Sweet is an Australian public health journalist and has been reporting on health-related topics for more than 30 years. She is Editor in Chief and a founding director of Croakey Health Media, a non-profit public interest journalism organisation with a focus on health equity, and curates a rotated Twitter account, @WePublicHealth. Dr Sweet holds an honorary appointment as Senior Lecturer at the Sydney School of Public Health at the University of Sydney and completed a PhD at the University of Canberra in 2017. Dr Sweet was asked as a panel member to contribute her expertise in public interest journalism.

Dr Penny Howard, National Research Officer with the Maritime Union of Australia (MUA)

The Maritime Union of Australia (MUA) represents maritime and dock workers nationally. Dr Penny Howard holds a PhD and is the National Research Officer at the MUA and conducts research and develops policy for the union and its membership. During the 2019-2020 bushfire season, Dr Howard and the MUA faced new challenges of providing health and safety advice to its members. The members are an outdoor occupational group which was especially impacted by the bushfire smoke episodes because work is strenuous, involves a high degree of communication among workers (which makes mask wearing difficult), is high pressure and cannot be moved indoors. Dr Howard was invited as a panel member to provide a perspective on outdoor occupational exposure to air pollution from bushfire smoke.
